# Comparative genomics of Australian isolates of the wheat stem rust pathogen *Puccinia graminis* f. sp. *tritici* reveals extensive polymorphism in candidate effector genes

**DOI:** 10.3389/fpls.2014.00759

**Published:** 2015-01-08

**Authors:** Narayana M. Upadhyaya, Diana P. Garnica, Haydar Karaoglu, Jana Sperschneider, Adnane Nemri, Bo Xu, Rohit Mago, Christina A. Cuomo, John P. Rathjen, Robert F. Park, Jeffrey G. Ellis, Peter N. Dodds

**Affiliations:** ^1^Agriculture Flagship, Commonwealth Scientific and Industrial Research OrganizationCanberra, ACT, Australia; ^2^Research School of Biology, Australian National UniversityCanberra, ACT, Australia; ^3^Plant Breeding Institute, Faculty of Agriculture and Environment, The University of SydneyNarellan, NSW, Australia; ^4^Genome Sequencing and Analysis Program, Broad Institute of MIT and HarvardCambridge, MA, USA

**Keywords:** haustoria, avirulence, resistance, secreted proteins, effectors

## Abstract

The wheat stem rust fungus *Puccinia graminis* f. sp. *tritici* (*Pgt*) is one of the most destructive pathogens of wheat. In this study, a draft genome was built for a founder Australian *Pgt* isolate of pathotype (pt.) 21-0 (collected in 1954) by next generation DNA sequencing. A combination of reference-based assembly using the genome of the previously sequenced American *Pgt* isolate CDL 75-36-700-3 (p7a) and *de novo* assembly were performed resulting in a 92 Mbp reference genome for *Pgt* isolate 21-0. Approximately 13 Mbp of *de novo* assembled sequence in this genome is not present in the p7a reference assembly. This novel sequence is not specific to 21-0 as it is also present in three other *Pgt* rust isolates of independent origin. The new reference genome was subsequently used to build a pan-genome based on five Australian *Pgt* isolates. Transcriptomes from germinated urediniospores and haustoria were separately assembled for pt. 21-0 and comparison of gene expression profiles showed differential expression in ∼10% of the genes each in germinated spores and haustoria. A total of 1,924 secreted proteins were predicted from the 21-0 transcriptome, of which 520 were classified as haustorial secreted proteins (HSPs). Comparison of 21-0 with two presumed clonal field derivatives of this lineage (collected in 1982 and 1984) that had evolved virulence on four additional resistance genes (*Sr5*, *Sr11*, *Sr27*, *SrSatu*) identified mutations in 25 HSP effector candidates. Some of these mutations could explain their novel virulence phenotypes.

## INTRODUCTION

Wheat stem rust, caused by *Puccinia graminis* f. sp. *tritici* (*Pgt*), is one of the most destructive diseases of wheat, barley and triticale (Leonard and Szabo, 2005; Park, 2007). In order to infect plants and cause disease, pathogens such as *Pgt* need first to overcome or evade the natural defenses of the plant. These defenses include preformed barriers, such as the waxy cuticle and inducible responses triggered by the plant innate immunity system (Jones and Takemoto, 2004). The first layer of the immune system involves recognition of pathogen associated molecular patterns (PAMPs) such as chitin or flagellin (Jones and Dangl, 2006; Dodds and Rathjen, 2010). Recognition of these factors by cell surface receptors leads to PAMP-triggered immunity (PTI), which is effective in preventing infection by non-adapted pathogens. Bacterial pathogens of plants overcome these defenses through the use of effector proteins that are delivered into host cells by a type III secretion system (Zhou and Chai, 2008), and biotrophic fungi and oomycetes also deliver effectors into host cells during infection (Giraldo and Valent, 2013). However, many of these effectors are recognized by a second layer of the plant defense system that involves intracellular receptors that are the products of the classically defined resistance (*R*) genes of the gene-for-gene system, first elucidated in the flax/flax rust pathosystem (Flor, 1971). In this context pathogen effectors are known as Avirulence (Avr) proteins and their recognition leads to rapid activation of a localized cell death termed the hypersensitive response, which is thought to limit the spread of the pathogen from the infection site (Chisholm et al., 2006). This second layer of defense has been termed effector-triggered immunity (ETI), and involves direct or indirect recognition of pathogen effector proteins by plant R proteins. Pathogens may evade this recognition by mutation of the corresponding *Avr* genes.

Many biotrophic fungi and oomycetes share a common infection process that involves the formation of haustoria, which invaginate and engage in close physical contact with the plasma membrane of host cells (Koeck et al., 2011). Haustoria play a role in nutrient acquisition and metabolism (Hahn and Mendgen, 2001; Voegele and Mendgen, 2003) and there is evidence to suggest that these structures also play a crucial role in the delivery of virulence effectors that alter defense responses and promote infection (Kemen et al., 2005; Whisson et al., 2007; Rafiqi et al., 2010). For example, all Avr genes that have been identified in the flax rust fungus (*Melampsora lini*) encode small secreted proteins that are expressed in haustoria and are recognized inside host cells by nucleotide binding leucine-rich repeat (NB-LRR) receptors (Dodds et al., 2004; Catanzariti et al., 2006; Barrett et al., 2009; Rafiqi et al., 2010). Analyses of transcript sets from isolated haustoria of *M. lini* (Nemri et al., 2014), the stripe rust pathogen *Puccinia striiformis* f. sp. *tritici* (*Pst*; Cantu et al., 2013; Garnica et al., 2013), common bean rust *Uromyces appendiculatus* (Link et al., 2013) and soybean rust *Phakopsora pachyrhizi* (Link et al., 2013) have predicted large numbers of secreted proteins expressed in these cells, indicating that they may deliver a large set of effectors to infected host cells. In the case of the wheat stem rust pathogen, whole genome shotgun sequencing of the American *Pgt* isolate CDL 75-36-700-3 (referred to as p7a) yielded an 81.5 Mbp genome sequence (out of an estimated 88.6 Mbp scaffold assembly) predicted to contain 15,979 protein coding genes (Duplessis et al., 2011). Of these about 10% are predicted to be secreted proteins, but their expression in haustoria has not been determined.

On the host side, there are more than 50 race-specific stem rust resistance *(Sr)* genes described in wheat, either derived from this species or introgressed from its close relatives (McIntosh et al., 1995), many of which have been deployed in modern wheat cultivars to control this disease. However, resistance breakdown has occurred frequently due to mutations in existing local isolates and the emergence or migration of new isolates, such as the highly virulent *Pgt* race Ug99 (Stokstad, 2007). In some areas where the alternate host of *Pgt* (*Berberis vulgaris*) exists, sexual recombination can give rise to new virulence phenotypes. Successful control of stem rust in wheat requires constant identification of new *Sr* genes, stacking of several different *Sr* genes in cultivars, and cultural efforts to keep inoculum levels low within each geographical zone of cultivation. The two recently cloned stem rust resistance genes *Sr33* (Periyannan et al., 2013) and *Sr35* (Saintenac et al., 2013) encode classical NB-LRR type intracellular immune receptors, suggesting that, as in *M. lini*, the corresponding *Pgt* Avr proteins are likely to be effectors delivered into host cells.

In Australia, there have been at least four independent incursions of exotic stem rust isolates documented since 1925. After arrival, the four founding isolates have each evolved mainly asexually in the field through presumed stepwise mutations that overcome various *Sr* genes deployed in wheat, leading to four clonal lineages comprising many derivative mutant pathotypes (pt.) differing for virulence on various host resistance genes (Park, 2007). In this study, we have used isolates of the four founder Australian *Pgt* pathotypes of these lineages and two mutant-derivative isolates of one lineage (pt. 21-0) with additional virulence, to construct the *Pgt* pan-genome, transcriptome and secretome. Comparisons of pt. 21-0 with the two presumed clonal field mutant derivatives with virulence to four additional resistance genes (*Sr5*, *Sr11*, *Sr27*, *SrSatu*) identified alterations in 25 haustorially-expressed effector candidates, which could include the mutations that give rise to their novel virulence phenotypes.

## MATERIALS AND METHODS

### *Puccinia graminis* f. sp. *tritici* (*Pgt*) ISOLATES

Individual isolates of four Australian *Pgt* (**Table 1**) pathotypes, 21-0 (Univeristy of Sydney accession number 54129), 126-5,6,7,11 (accession number 334), 194-1,2,3,5,6 (accession number 691042), and 326-1,2,3,5,6 (accession number 690822) were used in this study. Given that each is a specific isolate of a pathotype, and for simplicity, these are referred to as isolates 21-0, 126, 194, and 326 hereafter. Each isolate represents the original detection of four separate incursions of *Pgt* into Australia isolated from the field starting from mid 1920s that have been maintained as viable cultures in liquid nitrogen at Plant Breeding Institute, Cobbitty, NSW, Australia (Park, 2007). To ensure isolate purity, a single pustule from a low density infection was isolated from each isolate and propagated on wheat cultivar Morocco in isolation prior to DNA preparation. The identity and purity of each isolate was checked by pathogenicity tests with a set of host differentials. Two additional isolates, pathotypes 34-2,12 (accession number 82246) and 34-2,12,13 (accession number 84552; referred to as isolates 34M1 and 34M2 hereafter), were also purified from single pustules by growth on ‘Coorong’ (*Sr27*) and ‘Satu’ (*SrSatu*) triticale, respectively, and their identities and purity confirmed by pathogenicity analysis. Isolates 34M1 and 34M2 were collected from the field in 1982 and 1984, respectively. They are considered to be mutational derivatives of pt. 21-0, with added virulence for *Sr5*, *Sr11,* and *Sr27* (34M1) and *Sr5*, *Sr11*, *Sr27,* and *SrSatu* (34M2; Zwer et al., 1992). Both isolates were found to have SSR genotypes identical to isolate 21-0 when tested with 8 SSR markers (Zhang, 2008). These studies also demonstrated that isolates 126, 194, and 326 differed from each other, and from the 21-0/34M1/34M2 clade (Zhang, 2008).

**Table 1:** Australian *Pgt* isolates used in this study and their compatibility (*Avr*/*avr* profiles) with different host *R* genes.

*Pgt* isolate (short name)	Incursion/isolation year	Virulent	Avirulent	Mesothetic
126-5,6,7,11 (126)	1925	*Sr5, Sr7b,Sr8a, Sr8b, Sr15, Sr17*	*Sr6, Sr9b, Sr9e, Sr11, Sr21 Sr27, Sr30, Sr36, SrAgi, SrEM, SrSatu*	*Sr9g*
21-0	1954	*Sr7b, Sr9g*	*Sr5, Sr6, Sr8a, Sr8b,Sr9b, Sr9e, Sr11, Sr15, Sr17, Sr21 Sr27, Sr30, Sr36, SrAgi, SrEM, SrSatu*	
34-2,12 (34M1)	1982	*Sr5, Sr7b, Sr9g, Sr11, Sr27*	*Sr6, Sr8a, Sr8b, Sr9b, Sr9e, Sr15, Sr17, Sr21, Sr30, Sr36, SrAgi, SrEM, SrSatu*	
34-2,12,13 (34M2)	1984	*Sr5, Sr7b, Sr9g, Sr11, Sr27, SrSatu*	*Sr6, Sr8a, Sr8b, Sr9b, Sr9e, Sr15, Sr17, Sr21, Sr30, Sr36, SrAgi, SrEM*	
326-1,2,3,5,6 (326)	1969	*Sr6, Sr8a, Sr9b, Sr11, Sr17*	*Sr5, Sr7b, Sr8b, Sr9g, Sr9e, Sr15, Sr27, Sr30, Sr36, SrAgi, SrEM, SrSatu*	*Sr21*
194-1,2,3,5,6 (194)	1969	*Sr6, Sr7b, Sr8a, Sr9b, Sr11, Sr17*	*Sr5, Sr8b, Sr9g, Sr9e, Sr15, Sr21, Sr27, Sr30, Sr36, SrAgi, SrEM, SrSatu*

For rust infection, host plants were grown at high density (∼25 seeds per 12 cm pot with compost as growth media) to the two leaf stage (∼7 days) in a growth cabinet set at 18–25°C temperature and 16 h light. Spores (–80°C stock) were first thawed and heated to 42°C for 3 min, mixed with talcum powder and dusted over the plants. Pots were placed in a moist chamber for 24 h and then transferred back to the growth cabinet. For RNA isolation, infected plant leaves with high density pustules (1 or 2 days before sporulation) were harvested, snap frozen and stored at –80°C. For DNA isolation, mature spores were collected, dried and stored at –80°C.

### DNA ISOLATION FROM *Pgt* UREDINIOSPORES AND SEQUENCING

DNA was extracted from urediniospores by a CTAB extraction method (Rogers et al., 1989) with some modifications, including the use of 0.5 mm glass beads instead of fine sand and dry beating (2 × 1 min) at full speed on a dental amalgamator instead of grinding in liquid nitrogen. Extraction was carried out in several batches each with ∼50 mg of dry spores and equal volume of 0.5 mm glass beads to accumulate sufficient quantities of DNA from different isolates. After CTAB extraction, samples were treated with DNase-free RNAase, extracted with phenol/chloroform/isoamyl alcohol (25:24:1) and purified using Qiagen Genomic tips (cat No 10233, Qiagen). DNA quality was assessed using the Bioanalyzer 2100 (Agilent Technologies). Each 50 mg batch of spores yielded ∼20 ug of crude DNA, but the recovery from the Qiagen Genomic Tips was usually very low (∼15–20%) and so several batches were needed to amass sufficient genomic DNA for sequencing.

*Pgt* isolate 21-0 genomic DNA was sequenced by Roche GS FLX 454 technology at the Australian Genome Research Facility Ltd (AGRF – Australia). A 454 sequencing library was prepared from 5 ug of DNA using the GS General Library preparation kit (Roche Diagnostics). The library fragment size range was 300–500 bp. This library was processed using the GS emPCR and GS FLX LR70 Sequencing kits (Roche Diagnostics) and sequenced in the GS FLX machine. The sequence (fasta format.fna) and the quality score (.qual) outputs were used for further analysis as detailed later in the section.

DNA from urediniospores of isolates *Pgt* 21-0, 126, 194, and 326 were also sequenced using the Illumina GAII platform at the Broad Institute (75 bp paired-end reads). Image analysis and base calling (including quality scoring) were performed using Illumina’s Pipeline Analysis Software v1.4 or later. Genomic DNA from mutant isolates 34M1 and 34M2 was sequenced on the Illumina HiSeq platform at AGRF. Libraries were prepared with Agencourt SPRIworks System1 (Beckman Coulter Genomics) using Illumina paired-end library adaptors. Fragment sizes in the library ranged 248–578 bp (including adaptors). Library clusters were generated with the automated cBot system using the Illumina TruSeq PE Cluster Synthesis v2.0 kit and sequenced (100bp paired-end reads) in HiSeq2000 using Illumina TruSeq v2.0 kits. Image intensities and quality scored base calls were performed by the built in HiSeq Control Software and fed into further analysis pipeline as detailed later in the section. Raw sequence reads generated and used in this study are being submitted to NCBI and will be associated with BioProject PRJNA253722^1^.

### HAUSTORIAL ISOLATION

Twenty grams of infected wheat leaves (isolate 21-0, 10-days post-infection) were sequentially washed with chilled tap water, 2% bleach, water, 70% ethanol, and Milli-Q purified water. Initial stages of haustorial isolation were performed as described previously (Catanzariti et al., 2011) using a final 20-μm pore size nylon mesh to remove the bulk of the plant cell material. Further processing was performed by Percoll gradient fractionation as described previously (Garnica and Rathjen, 2014). Briefly, the filtrate was centrifuged at 1080 *g* for 15 min and the resulting pellet was resuspended in 80 ml of suspension buffer (Percoll 30%, 0.2 M sucrose, 20 mM MOPS pH 7.2). The suspension was divided into five tubes and then centrifuged at 25,000 *g* for 30 min. The top 10 ml of each tube was recovered, diluted 10 times with isolation buffer (0.2 M Sucrose, 20 mM MOPS pH 7.2) and centrifuged at 1,080 *g* for 15 min. The pellets were resuspended in suspension buffer with Percoll 25% and taken to a second round of isolation. The final pellet was frozen in liquid nitrogen and stored at –80°C prior to RNA isolation.

### RNA ISOLATION AND SEQUENCING

RNA was isolated from purified haustoria and spores germinated for 15 h on sterile distilled water (16°C in the dark). Samples were ground to a fine powder in liquid nitrogen and total RNA isolated using the RNeasy Plant Mini Kit (Qiagen). Extracted RNA was treated with RNase-free DNAse (Promega) and repurified using the RNeasy Plant Mini Kit columns. RNA quality was assessed with the Bioanalyzer 2100. About 10 μg of total RNA was processed with the mRNA-Seq Sample Preparation kit from Illumina to produce the sequencing libraries. Quality and quantity controls were run on an Agilent 2100 Bioanalyzer using a DNA 1000 chip kit and each library was diluted and used for sequencing with an Illumina Genome Analyser GX II platform (100 bp paired-end reads).

### GENOME AND TRANSCRIPTOME ASSEMBLY AND ANALYSES

**FIGURE 1:**
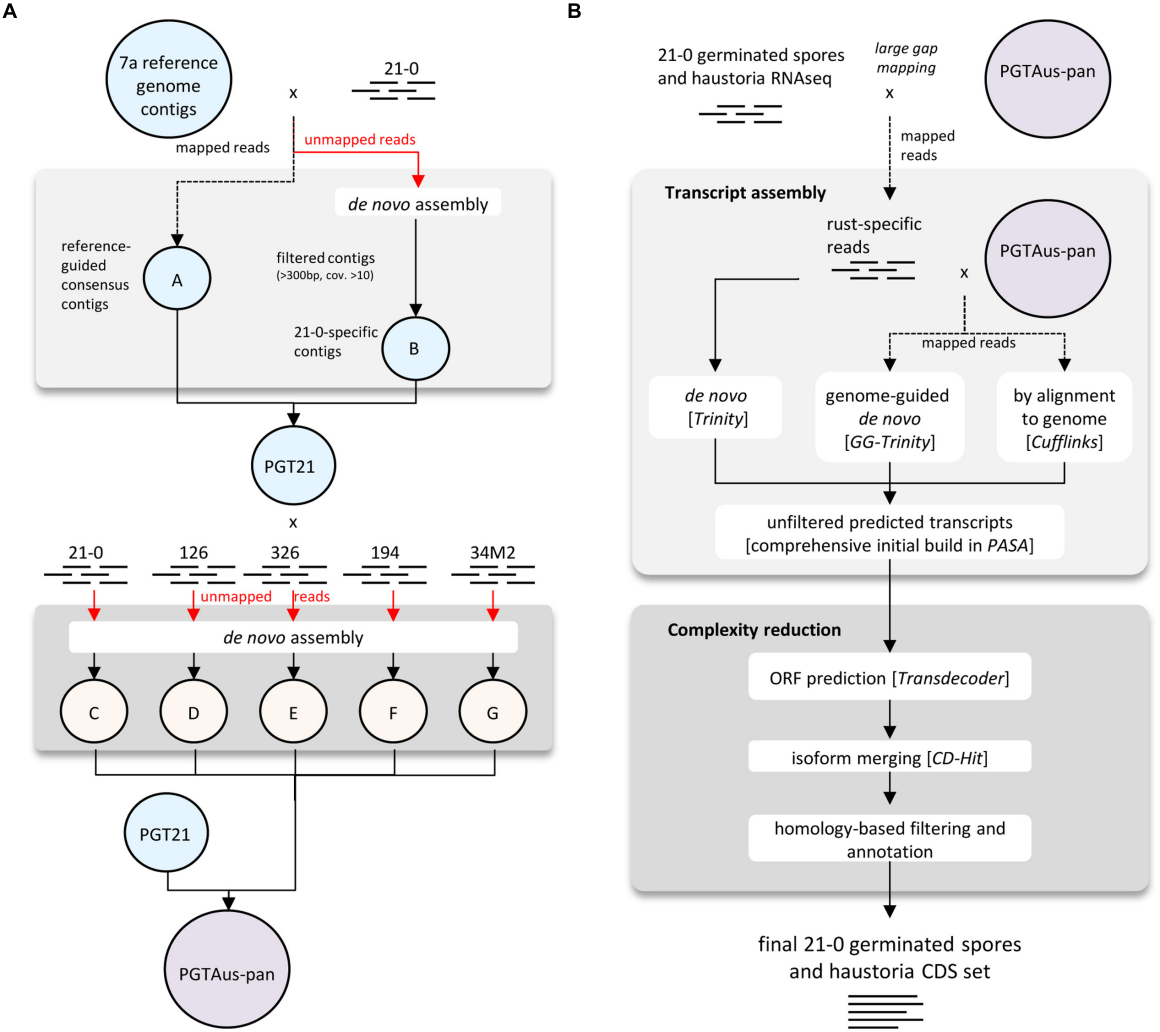
**Flow chart diagrams illustrating genome and transcriptome assembly pipelines. (A)** Pipeline for assembly of PGT21 and PGTAus-pan genomes from DNA reads from Australian *Pgt* isolates. **(B)** Pipeline for assembly of isolate 21-0 transcriptome from RNA reads from isolated haustoria and germinated spores.

A consensus reference genome was built using various modules available in CLC Genomics Workbench (Version 4.5 or later, CLC bio Qiagen, Prismet) and the analysis workflow as depicted in **Figure 1A**. Combined 454 and Illumina sequencing reads from isolate 21-0 DNA were first pre-processed (quality trim 0.01, adaptor trim, minimum length 40 nt, maximum ambiguity 2 nt, terminal trim 1 nt). Read mapping was performed using the CLC module Map Reads to Reference (default parameters) and the 4557 contigs of the *Pgt* isolate CDL 75-36-700-3 (Duplessis et al., 2011)^2^ as a reference for assembly. Consensus sequences derived from this mapping were taken as part A of our PGT21 reference build. The unmapped reads were assembled *de novo* and contigs of length >300 nucleotides and average coverage >10 were *de novo* assembled in a second round using ‘simple assembly,’ and added as part B of the PGT21 reference. DNA reads from isolates 21-0, 126, 194, 326, and 34M2 were mapped to PGT21 and the unmapped reads were *de novo* assembled separately and contigs >300 nucleotides and average coverage >10 (parts ‘C,’ ‘D,’ ‘E,’ ‘F,’ ‘G’ respectively) were added to the PGT21 assembly to generate the pan-genome assembly, PGTAus-pan.

For transcriptome assembly, quality trimmed (0.01 quality trim, minimum length 50) RNA reads from isolated haustoria and germinated spores from isolate 21-0 were first aligned to PGTAus-pan genome by using the CLC module large gap read mapping (default parameters) and mapped reads were extracted as fungal specific reads. Transcript models were built separately using genome-guided and *de novo* assembly with the Trinity pipeline (Grabherr et al., 2011) and a genome reference based assembly using Tophat/Cuﬄinks (Trapnell et al., 2012). These transcript models were used as inputs to the PASA (Program to Assemble Spliced Alignments) pipeline^3^ to build a comprehensive transcriptome database. Open reading frame (ORF) and protein predictions (>50 amino acids) were performed using Transdecoder^4^. A further complexity reduction was then performed on a non-redundant protein set with CD-hit (Li and Godzik, 2006) for isoform/allele merging^5^ (95% identity cut-off), yielding 27,150 proteins, which were reduced to 22,391 after manual curation to exclude likely spurious ORFs. Predicted proteins were analyzed for homology to known proteins (e-value 1e-20 cut-off) by PFAM domain searching (Punta et al., 2012) and by Blastp analysis (e-value 1e-05 cut-off) against a custom database of predicted proteins from *Pgt*, *Pst* (Cantu et al., 2013; Garnica et al., 2013) and *P. triticina*^6^. The presence of signal peptides was predicted using SignalP v4.0 using the SignalP-TM network function (Petersen et al., 2011). Transmembrane domains were then predicted using TmHMM (Krogh et al., 2001) and those proteins containing one or more transmembrane domains that did not overlap with the signal peptide (minimum five amino acids) were excluded from the secreted protein set. The 21-0 haustorial and germinated spore transcript models (coding sequences only) and the p7a reference transcript set (coding sequences only; 15,979 entries downloaded on 1-5-2014 from *Puccinia* Group Sequencing Project, Broad Institute of Harvard and MIT^7^) were mapped onto PGTAus-pan using the PASA pipeline.

### SNP DETECTION AND INTER-ISOLATE COMPARISONS

Unless otherwise mentioned, analysis was performed using programs and plug-ins available in CLC Genomics Workbench (V. 6.5.1 or later). Quality trimmed DNA reads (quality 0.01, minimum length 50 nt, adapter trimmed, and overlapping paired-end read merging) were mapped (default settings) to the annotated PGTAus-pan reference genome. Local realignments were performed before making variant calls using Probabilistic Variant Detection, ignoring non-specific matches and broken pairs and with default parameters including minimum coverage 10, variant probability 90% and minimum variant count 2. Variant comparison tables were produced and exported as VCF or CSV files for further processing. For assigning variants to coding and non-coding sequences, we used the combined p7a and 21-0 transcript annotation and chose the longest predicted coding sequence at each locus. To infer phylogenetic relationships between the sequenced isolates, variant calls were first filtered using custom Python scripts for homozygous SNPs (indels were ignored) and then merged and converted to tabular format using VCFtools (Danecek et al., 2011). From this, SNP alignments were concatenated and used as input to FastTree (Price et al., 2010), with the –pseudo and –nt options. Phylogenetic trees were drawn and midpoint rooted using MEGA6 (Tamura et al., 2013).

### GO ANNOTATION OF THE PREDICTED PROTEOME

For the gene ontology (GO) classification the set of 22,391 predicted genes was analyzed using the BLAST2GO PRO plugin in CLC genomics 6.5. Briefly, a Blastp search of predicted protein sequences against the non-redundant protein database (nr) of NCBI (Database downloaded on August-2013) was performed with a maximum expectation value of 1.0e-25, maximum number of alignments to report = 50 and highest scoring pair length = 33 amino acids. The GO terms associated with each BLAST hit were retrieved and GO annotation assignment to the query sequences was carried out using default parameters. BLAST2GO was also used for GO functional enrichment analysis of the genes differentially expressed in both germinated spores and haustoria, by performing Fisher’s exact test with false discovery rate (FDR) correction to obtain an adjusted *p*-value (0.05).

## RESULTS AND DISCUSSION

### GENOME ASSEMBLY OF AUSTRALIAN *Pgt* ISOLATES

To investigate genetic variation amongst Australian stem rust isolates, four isolates (21-0, 126, 194, and 326) with different virulence/avirulence phenotypes on the *Sr* resistance genes represented in standard differential genotypes (**Table 1**) and representing the four independent incursions of stem rust into Australia (Park, 2007) were each analyzed by next generation sequencing. Illumina sequencing (75 bp paired ends) data from genomic DNA of isolates 21-0, 126, 194, 326 yielded 41-178 million reads after quality-based filtering (**Table 2**) that were mapped to the 81.5 Mbp reference genome (4,557 contigs, 81,521,292bp) of the American *Pgt* isolate CDL 75-36-700-3 (p7a; Duplessis et al., 2011). Between 61 and 73% of the sequence reads for each isolate could be mapped to the p7a reference genome, covering between 94.8 and 97.6 of the reference at depths of 23- to 108-fold (**Table 2**). Mapped regions in isolates 21-0, 126, and 194 showed >98% sequence identities to the p7a reference, while isolate 326 was more divergent with only 93% identity.

**Table 2:** Mapping of Illumina DNA reads from Australian *Pgt* isolates against p7a reference.

	Pgt Isolates			
	21-0	194	326	126
Total reads (quality trimmed)^*^	178,487,947	124,005,114	41,202,425	134,392,144	
Reads mapped to reference	131,084,929	84,503,934	25,300,892	88,653,681	
Percentage mapped reads	73.44	68.15	61.41	65.97	
Total bases mapped to reference	8,556,866,766	5,558,168,397	1,823,251,337	5,533,033,972	
Assembly length (bp)	78,726,070	78,918,599	77,273,144	79,579,366	
Average times coverage	108.69	70.43	23.59	69.53	
Unmapped reads	47,403,018	39,501,180	15,901,533	45,738,463	
Percentage unmapped reads	26.56	31.85	38.59	34.03	
Percentage coverage of reference	96.57	96.81	94.79	97.62	
Percentage bases identical to reference^∗∗^	98.26	98.20	93.30	98.50	
Percentage mismatched bases**	1.11	1.14	1.15	0.91	
Percentage reference gap bases**	3.71	3.45	5.10	2.77	
Percentage assembly gap bases**	0.59	0.57	0.50	0.56


^*^CLC genomics workbench 4.9 or above was used for assembly (parameter settings: quality clip 0.05; conflict resolution by vote; random mapping of non-specific reads, two ambiguities allowed, read length 40–75 bp).

^**^Based on BLAT analysis between p7a contigs and respective assembled sequences.

For each isolate more than 25% of the reads did not map to the p7a reference, suggesting that these genomes contained substantial amounts of DNA sequence not present in the p7a reference genome. Therefore, we built a new reference genome based on the sequence of isolate 21-0 (**Figure 1A**; Table S1). We obtained additional 454 sequence data for this isolate (3 million reads, 1.2 Gbp, 12X coverage, average read length 400 bp). This sequence was combined with the Illumina sequence data and first assembled against the p7a reference genome. The consensus sequences for the 4,557 contigs in this assembly were then taken as part A of our PGT21 reference build (79.2 Mbp). The remaining unmapped reads were assembled *de novo* and contigs of length >300 nucleotides and average coverage >10X (19,662) were retained and again *de novo* assembled, resulting in a total of 16,960 contigs (part B, 13.3 Mbp), which were then added to the PGT21 reference build. The complete PGT21 genome assembly then comprised 21,517 contigs and ∼92.5 Mbp, about 11 Mbp larger than the p7a reference genome sequence. Much of this could represent sequence missing (gaps in the scaffold) from the p7a reference assembly, rather than isolate-specific sequence, because the p7a scaffold assembly size is 89 Mbp including gaps (Duplessis et al., 2011). The remainder of the additional sequence may represent highly variable regions between the two isolates that failed to map to the original reference sequence. The *de novo* assembled sequence region contained a similar density of heterozygous SNPs (see below) to the reference assembled region, indicating that it is present it both haploid nuclei, and does not represent a divergent sequence present in just one nucleus of this dikaryotic organism. Nearly 96% of the isolate 21-0 DNA reads could be remapped back to the PGT21 reference covering 99.35% of the assembly with an average nucleotide identity of 99.7% (**Table 3**).

**Table 3:** Mapping of Illumina DNA reads from Australian *Pgt* isolates to PGT21 reference genome.

	*Pgt* Isolates/mutants					
	21	34M1	34M2	194	326	126
Total reads (quality trimmed)*	155,272,002	312,359,971	165,949,995	106,502,558	24,201,578	106,119,468
Reads mapped to reference	148,738,543	292,860,382	160,656,060	98,176,567	23,470,521	93,092,615
Percentage mapped reads	95.79	93.76	96.81	92.18	96.98	87.72
Total bases mapped	9,324,322,642	28,576,013,167	15,032,224,237	5,981,339,426	1,166,889,391	5,398,916,769
Assembly length (bp)	91,842,155	90,688,020	90,159,598	91,123,290	89,660,526	88,928,858
Average times coverage	95	303	160	61	12	57
Unmapped reads	6,533,459	19,499,589	5,293,935	8,325,991	731,057	13,026,853
Percentage unmatched reads	4.05	6.24	3.19	7.82	3.02	12.28
Assembled contigs	21,517	21,517	21,192	21,513	21,509	21,513
Percentage coverage of reference	99.25	98.00	97.59	98.48	96.90	96.10
Percentage coverage of reference part B	∼100	99.23	96.98	98.26	96.34	86.57
Percentage bases identical to reference**	99.7	99.4	99.36	99.41	99.38	98.12
Percentage mismatched bases**	0.20	0.46	0.49	0.41	0.49	1.07
Percentage Reference gap bases**	0.72	1.83	2.1	1.46	2.79	3.79
Percentage Assembly gap bases**	0.10	0.13	0.13	0.16	0.12	0.65

*^∗^CLC genomics workbench 4.9 or above was used for assembly (parameter settings: quality limit 0.01, ambiguity allowed 1 nt from each end; length 25–77 nt except with 34M2 where length 50–100 nt; conflict resolution by vote; random mapping of non-specific reads).*

*^∗∗^Based on BLAT analysis between PGT21 and respective assembled sequences.*

Alignment of the DNA reads from the other Australian *Pgt* isolates (126, 194, 326), as well as from two additional isolates derived from 21-0 (34M1 and 34M2), to the PGT21 assembly showed that 88–97% of reads mapped to the PGT21 reference and covered 96–98% of the sequence and were at least 98.12% identical (**Table 3**). Sequence reads from the independent isolates 126, 194, and 326 covered between 87 and 98% of the additional 13.3 Mbp *de novo* assembled region of the PGT21 (part B), indicating that most of this region is not specific to isolate 21-0. To capture possible isolate-specific sequences from other Australian isolates, additional unmapped DNA reads from 21-0, 126, 194, 326, and 34M2 were *de novo* assembled independently and contigs >300 bp and >10x coverage were added to the PGT21 reference (Parts C to G respectively) to obtain the pan-genome PGTAus-pan (**Figure 1A**; Table S1). Isolate 126 showed the highest level of unique sequence (∼1%, Table S1), as well as the greatest number of mismatches, gaps and unmapped reads (**Table 3**), suggesting a greater evolutionary divergence of isolate 126 from 21-0 compared to the other isolates. This is consistent with previous studies of genetic diversity among Australian isolates of *Pgt* using other DNA marker systems (Keiper et al., 2003; Zhang, 2008). An analysis searching for the CEGMA set of 248 conserved eukaryotic genes (Parra et al., 2007) found that 237 (95.5%) were present in full or in part in the PGTAus-pan assembly, compared to 232 (93.5%) for the p7A reference genome, indicating an improvement in gene coverage in the PGTAus-pan genome compared with the p7a reference. Altogether, we have assembled a 92 Mbp *Pgt* pan-genome, which contains a significant amount of novel sequence not included in the p7a assembly. Most of this sequence is nevertheless common amongst several stem rust isolates, thus resulting in higher genome coverage for the wheat stem rust pathogen.

### ANNOTATION OF TRANSCRIPTS ON THE PGTAus-PAN GENOME

As a first step toward annotating the Australian *Pgt* pan-genome, the 15,979 transcripts (ORFs only) previously predicted for p7a were mapped against PGTAus-pan using the PASA pipeline (Haas et al., 2003). Under the set stringency (alignment length >75% and identity >95%) 14,843 transcripts aligned to the genome (Table S3) and as expected, almost all (14,828) mapped to part A of PGT21 (p7a reference assembled), while 15 transcripts mapped to other parts of the pan-genome. In total, 13,554 valid ORFs (>50 amino acids) could be predicted from the mapped transcripts.

For a more comprehensive annotation of the PGTAus-pan genome, we generated a new transcript set from RNA isolated from purified haustoria and germinated spores of isolate 21-0 as outlined in **Figure 1B**. Three biological replicate samples were sequenced by Illumina HiSeq 2000 (100 bp paired ends) to also allow subsequent differential expression analysis (see below). Initial raw reads (∼25 million pairs in each replicate sample) yielded 17-23 million quality-trimmed pairs per replication (Table S2). Large-gap read mapping (CLC Genomics Workbench) to PGTAus-pan was used to extract *Pgt*-specific reads. Transcripts were built independently by three methods using pooled reads: trinity assembly using both *de novo* and genome-guided approaches and TopHat/Cuﬄinks assembly against the PGTAus-pan reference. Transcripts from these independent assemblies were combined and assembled using the PASA pipeline to give a comprehensive initial transcriptome set of 61,451 transcript models. Of these, 59,783 could be aligned to the genome with 55,386 correctly mapping to predicted exon boundaries (Table S3). Most of these (85.6%) mapped to the p7a reference-assembled region (part A) of the PGT21 genome, while 13.4% mapped to the *de novo* assembled region (Part B). A small number (587, ∼1%) of transcripts mapped to other parts of the pan-genome (C to G).

A total of 22,391 non-redundant protein sequences were predicted from the transcript models after complexity reduction and filtering as described in the methods (Table S4). Approximately 90% (20,242) of these proteins mapped to PGT21 part A (i.e., common to PGT21 and p7a) and 9.3% (2,091) mapped to PGT21 part B. In a Blastp search against a custom database of predicted proteins from *Pgt*, *Pst*, and *P. triticina*, a previously annotated homolog in one of these *Puccinia* species was detected for 19,311 proteins (e-value 1e-05 cut-off). Interestingly, only 15,923 showed best Blastp hits to *Pgt* proteins while the remainder returned best hits to *Pst* (2,026) or *P. triticina* (1,381) proteins. These include genes that either were not present in the Pgt7a reference genome assembly (1,041 mapped to part B of PGT21) or were not annotated in the sequence (2,062 mapping to part A). A further 3,061 ORFs/proteins had no significant Blastp hit to the *Puccinia* group protein set but did align to PGT21 and may represent novel rust genes not previously detected. Only five transcripts failed to align to the PGTAus-pan sequence, and these showed significant hits to wheat cDNA sequences suggesting they are derived from host RNA contaminants. Another three transcripts with poor alignment to PGTAus-pan sequence had better matches to wheat transcripts. None of the other sequences appeared to be derived from wheat genes. We have also flagged 26 transcripts of possible *Pgt* mitochondrial origin. The PGTAus-pan genome sequence was annotated with the aligned transcripts from both p7a and the 21-0 transcriptome build^8^. A total of 21,874 gene loci are predicted in this annotation, which is similar to the gene numbers predicted for other rust fungal genomes such as *Melampsora larici-populina* (16,399, Duplessis et al., 2011), *M. lini* (16,271, Nemri et al., 2014), and *Pst* [20-25,000, (Cantu et al., 2013; Zheng et al., 2013)].

### COMPARISON OF HAUSTORIAL AND GERMINATED SPORE TRANSCRIPTOMES

**FIGURE 2:**
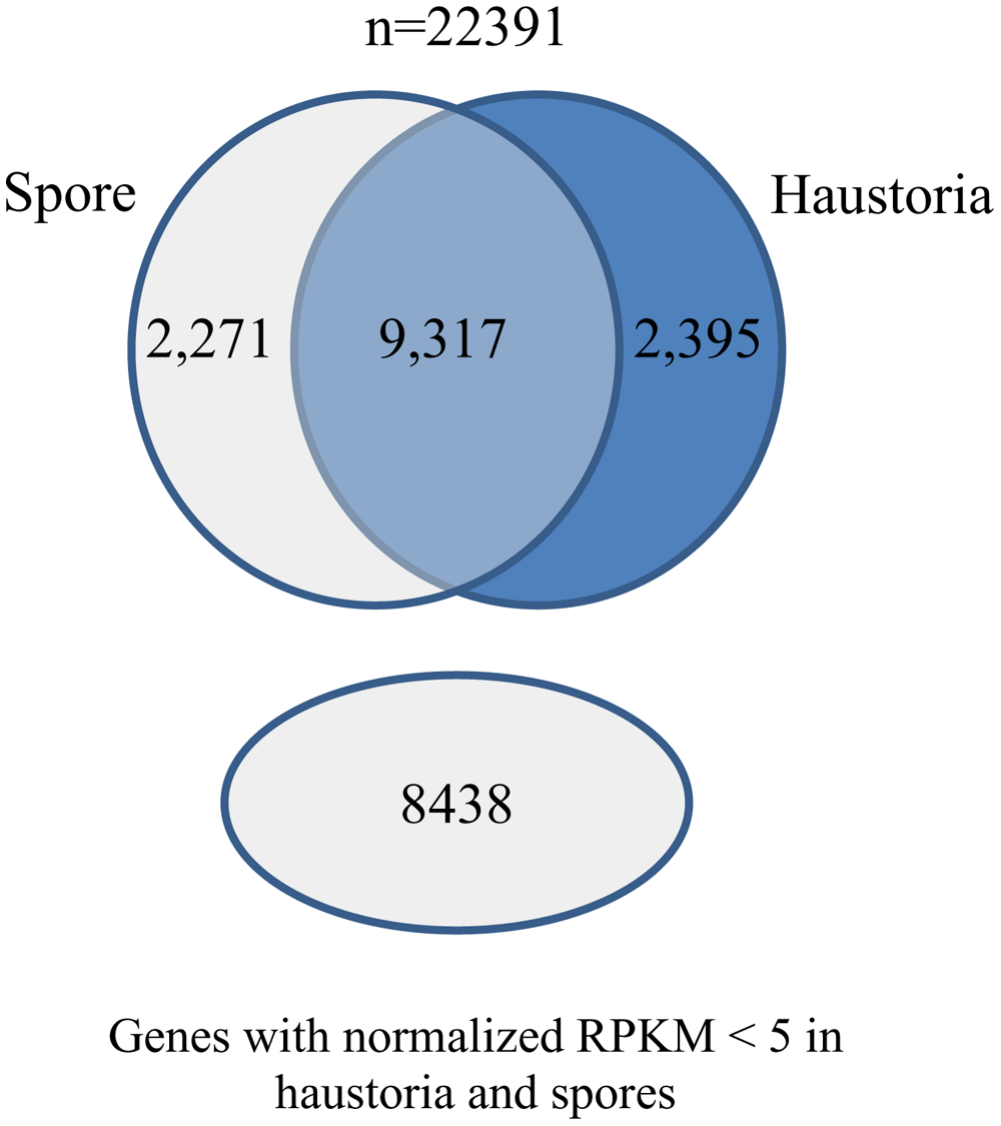
**Differential gene expression in *Pgt* germinated spores and haustoria.** Venn diagram of predicted gene set showing the number of genes that had statistically significant changes in expression between germinated spores and haustoria (Baggerley’s test FDR corrected *p*-value < 0.05 and expression difference > 20RPKM), genes that did not show differential expression between the two tissues, and those that showed very low expression (RPKM < 5) in both tissues.

We also used the RNA-Seq data from isolated haustoria and germinated urediniospores to compare gene expression between these cell types. The data were each obtained from three independent biological replicates allowing statistically robust quantitative expression analysis. The RNA-Seq tools from CLC genomics were used to align the raw Illumina reads against the reference transcript set and expression levels were quantified as reads per kilobase per million mapped reads (RPKM) for comparison of transcript levels. A total of 4,524 genes were differentially expressed between these cell types, with approximately half upregulated in haustoria and half in germinated spores (**Figure 2**).

**FIGURE 3:**
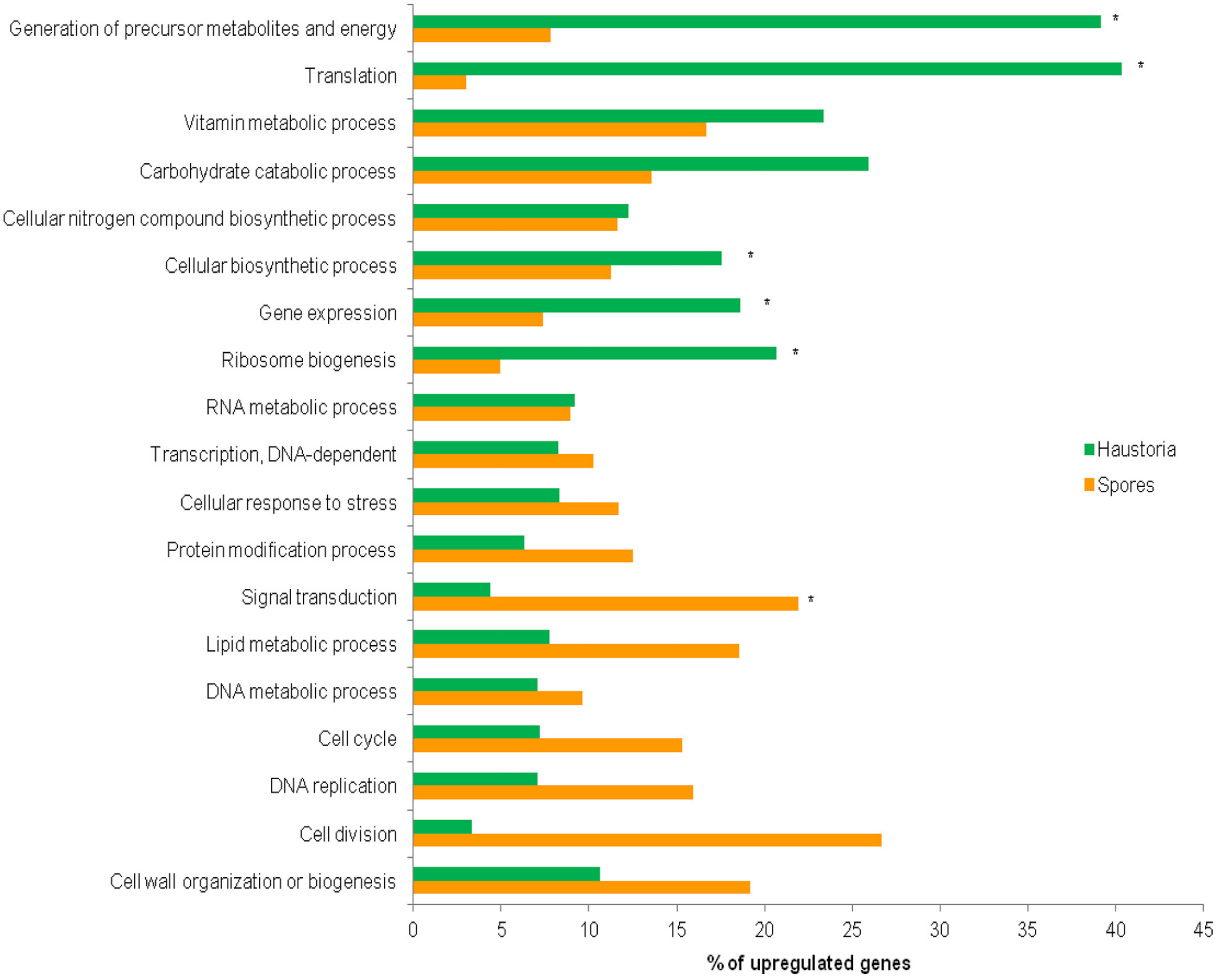
**Comparative ontology analysis of genes with statistically significant changes in expression between haustoria and germinated spores.** Of the original predicted gene set, 34.6% (794) of the 2,365 haustorial-enriched genes and 46.4% (1,037) of the 2,271 genes up-regulated in germinated spores were annotated with B2G. Relevant biological process GO terms are shown on the Y-axis. Percentages of genes differentially expressed in each tissue belonging to the nominated categories are shown on the X-axis. Asterisks indicate the categories over-represented in either developmental stage found after applying Fisher’s exact test, FDR < 0.05.

The 22,391 predicted gene set was annotated using BLAST2GO software (Conesa et al., 2005; Figure S1A). Among all Blastp results, *P. graminis*, *M. larici-populina, Cryptococcus neoformans*, *Agaricus bisporus,* and *Serpula lacrymans* were the top five species in terms of the total number of hits to the NCBI-nr protein database (Figure S1B). In total 7,469 (33.4%) genes could be unambiguously annotated with predicted functions and were categorized into functional classes to identify those that encode proteins with known roles in cellular processes. Direct GO count graphs were created to categorize the sequences to several groups based on their biological process ontologies (Table S5), the major functional categories are shown in **Figure 3**. Processes upregulated in germinated spores were representative of cell proliferation, such as cell cycle, DNA replication and cell wall biogenesis, whereas haustoria were committed to energy production and biosynthetic processes. Similar observations were recently made for the stripe rust pathogen *Pst* (Garnica et al., 2013). Other similarities with *Pst* included the upregulation of genes involved in the production of ATP through glycolysis, TCA cycle and oxidative phosphorylation in haustoria of *Pgt,* and upregulation of genes involved in releasing energy from stored lipid reserves and processing them via the glyoxylate/gluconeogenesis pathways in spores (Table S6). This suggests that the primary metabolism of haustoria and germinated spores of these two rust pathogens is largely the same. Recent transcriptomic studies on isolated haustoria from other rust fungi (Link et al., 2013) revealed important metabolic similarities to *Pgt* and *Pst*, supporting the idea that both the structure and the physiology of the haustorium are hallmarks of biotrophy in rust fungi.

**FIGURE 4:**
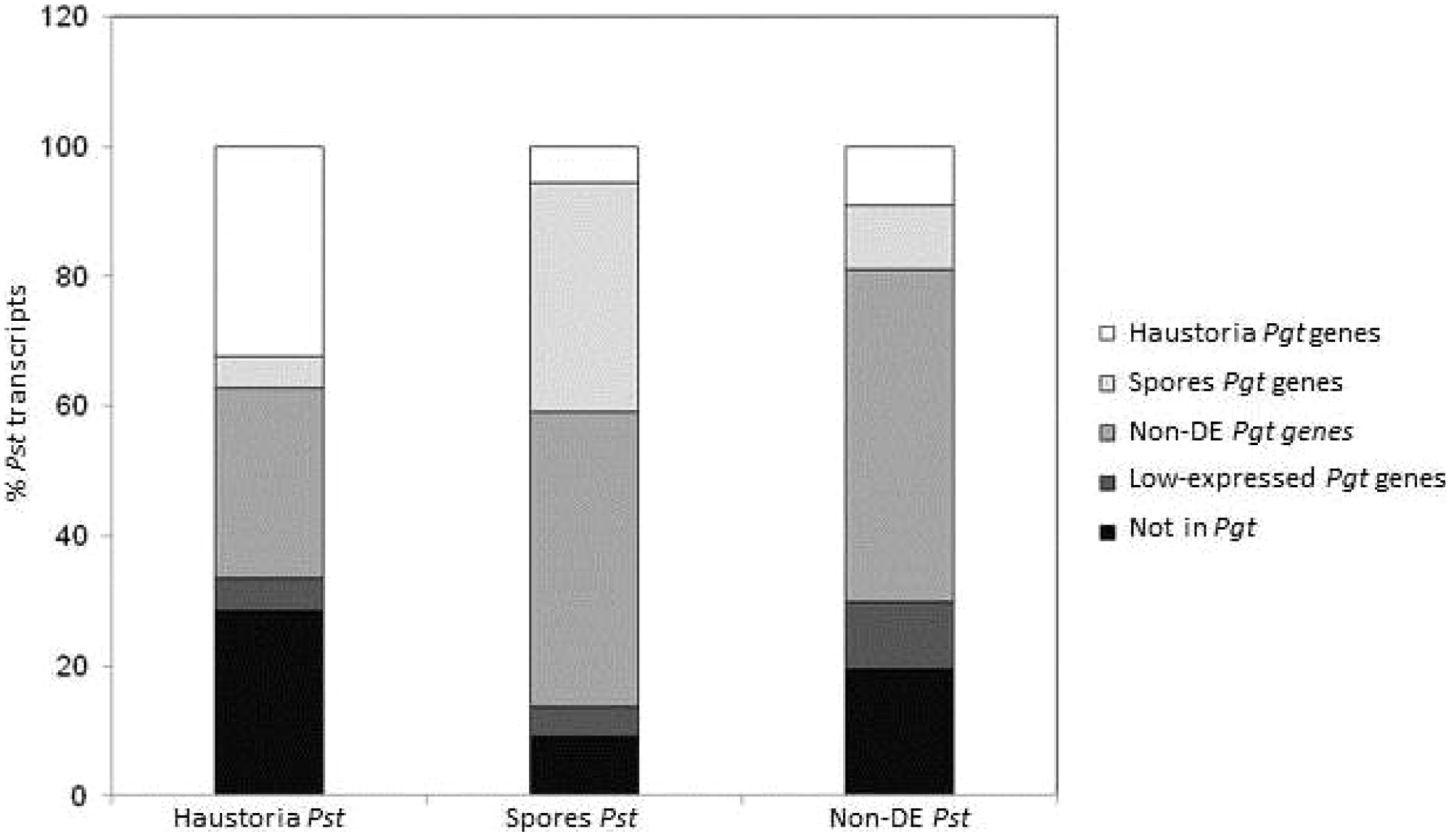
**Comparison of gene expression profiles in *Pgt* and *Pst* in haustoria and germinated spores stages.** A total of 12,282 transcripts from *Pst* previously classified accordingly to their statistically significant over expression in germinated spores (2,357) and haustoria (1,989), or those that did not show differential expression between the two tissues (Non-DE, 7,936) were BLAST searched (max E-val 1.0e-6) against the *Pgt* 21-0 predicted gene set. The percentage of transcripts matching genes in the same (or different) expression profile category in *Pgt* are shown in colors.

To determine broader similarities in the gene expression profiles between *Pgt* and *Pst*, the whole set of *Pgt* predicted genes was compared to transcriptomic data for *Pst* (Garnica et al., 2013). The 12,282 transcripts from *Pst* were BLAST searched against the predicted gene set of *Pgt* and then matched accordingly to their tissue expression profile (**Figure 4**). A total of 9,962 transcripts from *Pst* (81%) showed similarity (e-value 1e-5 cut-off) to at least one predicted gene from *Pgt*. Although only 56% of the matching genes had the same expression profile in both species, most of these differences were genes showing differential expression in one species but either not deferentially expressed or expressed at a low level in the other, probably mainly reflecting differences in the sensitivity of the statistical tests applied. Despite this, there was a broad similarity in the expression data for both species. *Pgt* homologs of *Pst* genes upregulated in haustoria were enriched for haustorial-specific genes, while *Pgt* homologs of *Pst* genes upregulated in spores were enriched for spore-expressed genes. Furthermore, most of the genes belonging to the metabolic categories mentioned above showed the same expression trends in both pathogens (**Figure 3**; Table S6).

### PREDICTION OF EFFECTOR CANDIDATES

To identify potential effectors in the PGT21 genome, we searched for proteins containing a predicted signal peptide (SP) in the haustorial and germinated spore transcript sets. Proteins containing one or more transmembrane domains (not overlapping with SP domain) were excluded, leaving a total of 1,924 predicted secreted proteins (Table S7). Of these, 1,590 had best Blastp hits to *Pgt* (p7a), while 103 and 81 had best hits in the *Pst* and *P. triticina* protein sets respectively, with the remaining 150 showing no hits. Of the 1,924 predicted secreted proteins, 1,824 were encoded in part A of PGT21, and 100 in part B. Over half (1,022) of these proteins have 4 or more cysteine (cys) residues while 212 have 10 or more cys residues, a common feature of many predicted and known effector proteins (Templeton et al., 1994).

Gene expression analysis detected 689 predicted secreted protein transcripts that were upregulated in haustoria (FDR corrected *p*-value < 0.05, >2 fold change, >5 normalized RPKM) while 460 were upregulated in germinated spores. Eliminating those with the lowest expression levels (<20 RPKM) left a set of 430 upregulated in haustoria and 329 in germinated spores. We considered the 430 haustorially upregulated secreted proteins as primary candidates for stem rust effectors. However, some rust effectors could also be expressed in germinated spores, as is the case for AvrM in flax rust (Catanzariti et al., 2006). Therefore we also considered those that showed high expression in haustoria (>100 RPKM) as good candidates regardless of their expression in germinated spores. This added an additional 90 genes to make a total set of 520 haustorial secreted proteins (HSPs). These included 299 proteins containing four or more cys residues and 85 with 10 or more. Only 41 of these could be annotated with putative function (PFAM hit with e-value < 1e-20), including seven carbohydrate-active enzymes, two heat shock proteins, two thaumatin-like proteins and three thioredoxin proteins (Table S7). Similar numbers of HSPs have been predicted from haustorial transcriptomes of *Pst* (437, Garnica et al., 2013) *U. appendiculatus* and *P. pachyrhizi* (395 and 149 respectively, Link et al., 2013).

### GENOME DIVERSITY BETWEEN ISOLATES

We examined genome-wide sequence variation both within and between the six Australian *Pgt* isolates by aligning the sequence reads from each isolate to the PGTAus-pan genome reference. For isolate 21-0, we found over 1.3 million variants, including single nucleotide variants (SNVs), multiple nucleotide variants (MNVs), and insertion/deletions (indels; Table S8A). These occurred at an overall frequency of 14.2/kb of mapped consensus sequence, with base changes (SNVs and MNVs) representing about 86% of this variation (12.3/kb). The vast majority of these variants (∼92%) occurred in a heterozygous condition, reflecting a high level of divergence between the two haploid nuclei in this dikaryotic organism. The frequency of variants was broadly similar in intergenic regions (13.37/kb), gene-coding regions (including introns, 15.88/kb) and coding sequences (12.79/kb), but there was a difference in the distribution of indels between these locations, being much more frequent in intergenic regions (14.3% of variation, 1.92/kb) than in coding sequences (5.4% of variation, 0.69/kb). A total of 153,946 (6.6/kb) variants could give rise to altered protein sequences including 136,516 non-synonymous SNPs (SNV+MNV) as well as 16,304 indels and these were distributed in 17,960 genes.

The frequency of DNA variation in the other isolates was similar to 21-0 (about 13-15/kb), except in isolate 326 where the low variant discovery (4.3/kb) may be attributed to the lower coverage of reads used in the initial mapping. As with 21-0, these isolates were heterozygous for the majority of variants (92–96%), except isolate 126, which contained a high proportion of variants that were in the homozygous state (43%). Thus, substantial variation between heterokaryons seems to be a common feature of *Pgt* isolates. Similarly, Cantu et al. (2013) found substantial polymorphism between heterokaryons in five isolates of *Pst*, with heterozygous SNPs occurring at a frequency of ∼6 per kb and representing over 90% of the total (homozygous and heterozygous) variation. Zheng et al. (2013) found a much lower rate of heterozygosity (∼1.0 SNP/kb) in *Pst* isolates, possibly because their genome assembly from fosmid clones resulted in separate assembly of allelic regions from the two haplotypes. Since *Pgt* reproduces asexually in Australia the heterozygosity present in these isolates, derived from their most recent sexual ancestor before incursion of these isolates into Australia, has been fixed. This is clearly observed in the case of the 34M1 and 34M2 which are clonally derived from 21-0 but isolated around 30 years after its incursion, and share almost all of the >1 million heterozygous SNPs that are present in 21-0. The high proportion of variant homozygosity in isolate 126 may reflect a level of inbreeding in the most recent sexual background of isolate 126, while 21-0, 194, and 326 may have arisen from more diverse populations.

**FIGURE 5:**
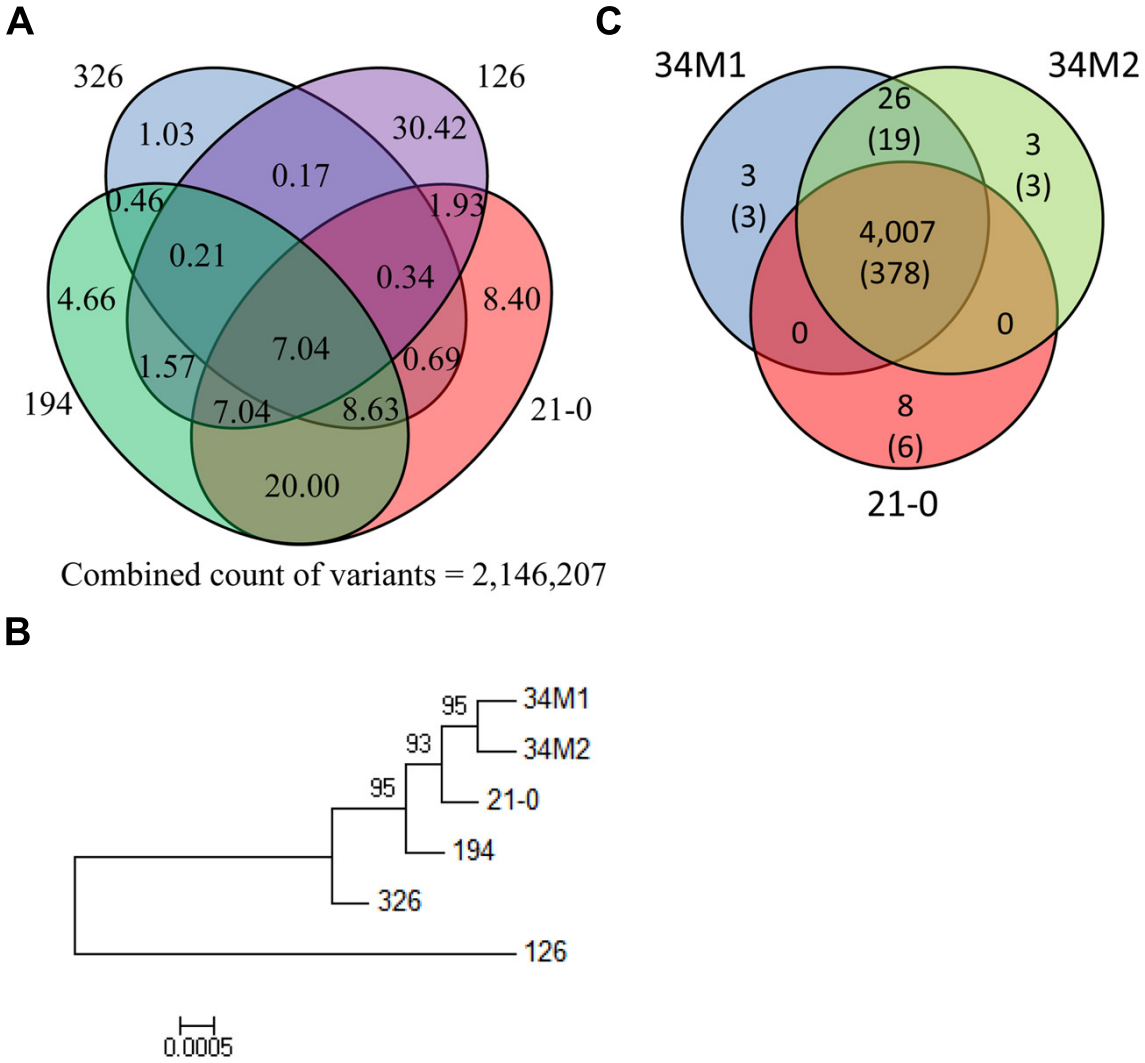
**Comparison of Australian *Pgt* isolate genome sequences. (A)** Venn diagram showing the percentage of the total number of polymorphisms detected in the four Australian founder *Pgt* isolates (21-0, 126, 194, and 326) that are shared between different isolates. **(B)** Phylogenetic tree of Australian *Pgt* isolates. This phylogeny was produced by FastTree from homozygous SNPs in the six stem rust isolates. Local bootstrap support values are shown. This tree is midpoint rooted. **(C)** Venn diagram showing numbers of shared non-synonymous polymorphisms in HSP genes between 21-0, 34M1 and 34M2. Figures in the parenthesis refer to the number of genes in which the polymorphisms occur.

To determine relationships between the *Pgt* isolates, we compared genome-wide variation between the isolates (**Figure 5A**). Variation between the four founder isolates was substantial: for instance only 7.0% of variation in 21-0 was shared with the other four isolates. In isolates 21-0, 194 and 326, unique variants were 8.4%, 4.7% and 1.0%, respectively, while isolate 126 was much more divergent with 30.4% unique variants. A phylogenetic tree constructed using the homozygous SNP data for the six isolates (**Figure 5B**), showed that isolates 34M1 and 34M2 fell into a clade derived from 21-0, consistent with the prediction that these isolates represent field-evolved mutational derivatives of 21-0 based on virulence phenotypes (Park, 2007). As noted above, isolate 126 showed greater divergence from the other isolates in this group.

### VARIATION IN EFFECTOR CANDIDATES

We examined variation in the set of 520 HSPs as these are most likely to include genes controlling virulence/avirulence differences between isolates with respect to infection on host differentials carrying different *Sr* genes. In 21-0, 402 (77%) of these genes contained sequence variants in their coding sequences (17.72/kb, almost all heterozygous), while 52 (∼10%) were not polymorphic and the remainder (∼13%) could not be scored due to incomplete mapping to the genome (Table S8B). In total, 3,843 variants (9.60/kb) occurring in 374 HSPs, gave rise to amino acid changes in the encoded proteins (including indels and frameshifts). Among the four Australian isolates, 16,322 variants were detected in 427 HSPs and showed a similar pattern of shared and unique polymorphisms as for the genome-wide variants (Figure S2). These included 5,245 non-synonymous variants in 406 genes. In a similar analysis of two UK *Pst* isolates that differ in only 2 virulence phenotypes, Cantu et al. (2013) found polymorphisms in 60 HSPs. However, this analysis only considered homozygous SNPs between the strains and heterozygous differences may account for significantly more differences. Bruce et al. (2014) observed much lower levels of diversity in effector candidates from *P. triticina*, with only 15 of 532 secreted proteins expressed *in planta*, showing amino acid differences among six isolates. However, this analysis was conducted using protein sequences translated from consensus-derived RNAseq transcripts and thus also does not consider heterozygous variation. The true extent of variation between these strains may be significantly higher.

As indicated previously, isolates 34M1 and 34M2 represent field-derived mutants of isolate 21-0 that have gained virulence for resistance genes *Sr5*, *Sr11,* and *Sr27* and in the case of 34M2 one additional *R* gene, *SrSatu*. We therefore examined nucleotide variants that give rise to altered amino acid sequences among the HSP set in these isolates. There were a total of 4,048 such nucleotide variants, of which the vast majority (3,712) were common to all three isolates. We manually examined the remaining 336 putative SNPs that distinguished the strains to eliminate any incorrect calls. In most cases reads representing each polymorphic variant were present in all three strains, although the SNP failed to be called in one or more strains. In only one case there was a false positive call. After manual curation, 4,007 SNPs were common to all three strains, while only 40 SNPs distinguished the strains (**Figure 5C**). Of these, 26 were common to 34M1 and 34M2 and absent in 21-0, and therefore represent novel mutations in these isolates that could explain their virulence on *Sr5*, *Sr11,* or *Sr27*. These occurred in a total of 19 HSP genes. The three variants (occurring in three genes) that were unique to 34M2 could explain virulence on *SrSatu*, giving a total of 22 candidates for these four *Avr* genes. We do not know whether the progenitor pathotype 21-0, is functionally homozygous at these *Avr* loci, in which case mutation of both alleles would be required for virulence, or heterozygous in which case a single mutation would be sufficient. In addition, eight variants (in six genes) were unique to 21-0. Loss of 21-0 variants could result from a deletion of one allele, but in all of these cases other heterozygous variants are retained in the HSP gene, ruling out this possibility. Alternatively, mutation of one variant site to the opposite allelic version could lead to virulence if the pathotype was heterozygous for this character. Thus these are also possible virulence mutations, giving a further three unique candidates for these *Avr* genes, for a total of 25 (Table S9). Clearly there are more HSP genes showing variation than the four documented *Avr* changes separating 34M2 from its progenitor 21-0. Mutations in other HSP genes that altered virulence-avirulence on uncharacterized *Sr* genes in wild host species may have been selected between the 1954 and 1984 isolations of 21-0 and 34M2. Furthermore, based on the strong assumption that effectors in *Pgt* play a virulence role, selection may occur in these genes for improved adaptation to host virulence targets in wheat or wild hosts. There may also be selection for changes in ‘background’ effector genes that compensate for loss of function in effectors associated with virulence-avirulence toward *Sr5*, *Sr11*, *Sr27,* and *SrSatu*.

## CONCLUSION

To summarize, we have generated an extended pan-genome for the wheat stem rust fungus that extends the previous reference assembly based on the p7a isolate by including about 13 Mbp of novel sequence. We carefully considered whether this additional sequence was specific to different strains, as substantial genome divergence has been observed for some other fungal plant pathogens. For instance, *Magnaporthe oryzae* strains contain up to 5% unique sequence that is dispersed throughout the genome (Yoshida et al., 2009), while *Fusarium oxysporum* contains several dispensable chromosomes that can vary in presence between strains infecting different hosts (Ma et al., 2010). Divergence between the haploid nuclei in the dikaryotic *Pgt* could also be a source of diversity in genome content. However, the vast majority of this additional sequence was represented in four unrelated isolates that each arrived in Australia at different times over the past century. The presence of heterozygous SNPs in this region also indicates that it is not derived from a single nucleus due to genome divergence between the haploid nuclei. Hence we suggest that most of this region is not strain specific, but more likely represents sequence that was simply not assembled in the p7a reference. Thus, the genome assembly presented here increases the sequenced genome coverage of this organism, improving the representation of core eukaryotic genes, and allowing the annotation of about 2000 transcripts in this region. Transcriptome assembly from germinated urediniospores and haustoria also identified a further ∼3500 transcripts not previously annotated in the p7a reference genome as well as a large number of potential alternative transcripts. Analysis of putative secreted proteins identified 520 HSPs as effector candidates, and a subset of 25 of these represent candidates for four *Avr* genes that differ between the pt 21-0 isolate and two derived isolates. We are currently performing functional analyses of these candidates by bacterial delivery to resistant host lines (Upadhyaya et al., 2014) to determine whether they encode these *Avr* recognition specificities. We are also selecting *de novo* mutants of 21-0 that acquire virulence toward *Sr5*, *Sr11*, *Sr27,* and *SrSatu* in glasshouse experiments so that sequence comparisons can be made between the candidate genes in 21-0 and the same genes in the new mutants.

## AUTHOR CONTRIBUTIONS

Narayana M. Upadhyaya, Diana P. Garnica, Adnane Nemri, Jana Sperschneider, Christina A. Cuomo, Haydar Karaoglu, Bo Xu, Rohit Mago performed experiments and analyzed data. Narayana M. Upadhyaya, John P. Rathjen, Robert F. Park, Jeffrey G. Ellis, Peter N. Dodds provided scientific direction. All contributed to the preparation of the manuscript.

## Conflict of Interest Statement

The Guest Associate Dr. David L. Joly declares that, despite having collaborated with author Christina A. Cuomo, the review process was handled objectively. The authors declare that the research was conducted in the absence of any commercial or financial relationships that could be construed as a potential conflict of interest.

## Footnotes

1 http://www.ncbi.nlm.nih.gov/bioproject/253722

2 http://www.broadinstitute.org/annotation/genome/puccinia_group/MultiHome.html

3 http://pasa.sourceforge.net/

4 http://transdecoder.sourceforge.net

5 http://weizhong-lab.ucsd.edu/

6 http://www.broadinstitute.org/annotation/genome/puccinia_group/MultiHome.html

7 http://www.broadinstitute.org/

8 http://webapollo.bioinformatics.csiro.au/puccinia_graminis_tritici_PGTAus-pan/index.html
