# Supplementary material for: Comparative genomics of Australian isolates of the wheat stem rust pathogen Puccinia graminis f. sp. tritici reveals extensive polymorphism in candidate effector genes
Source: Front Plant Sci. 2015 Jan 8;5:759. doi: 10.3389/fpls.2014.00759 (PMC4288056; doi:10.3389/fpls.2014.00759)
Supplement: Supplementary file 1 [file Data_Sheet_1.ZIP › Table_S9_30Sept2014.docx]

**Table S9.** Short listing of 25 HSPs as candidate effectors imparting avirulence on *Sr5*, *Sr11*, *Sr27* and *SrSatu.*

| Gene ID | Count of shared or unique variants | | | | | |
| --- | --- | --- | --- | --- | --- | --- |
|  | 210 | All 3 | 34M1 | 34M1, 34M2 | 34M2 | Total |
| HSGS210\|asmbl_10510\|m.1763 | 1 |  |  | 1 |  | 1 |
| HSGS210\|asmbl_12816\|m.8565 |  | 2 |  | 1 |  | 3 |
| HSGS210\|asmbl_14320\|m.14022 |  |  |  | 1 |  | 1 |
| HSGS210\|asmbl_15108\|m.17363 |  |  |  | 1 |  | 1 |
| HSGS210\|asmbl_19943\|m.35422 | 1 |  |  |  |  | 1 |
| HSGS210\|asmbl_19979\|m.35585 |  | 1 |  | 1 |  | 2 |
| HSGS210\|asmbl_21481\|m.41346 | 1 |  | 1 | 1 |  | 3 |
| HSGS210\|asmbl_22318\|m.44511 |  |  |  |  | 1 | 1 |
| HSGS210\|asmbl_25911\|m.58390 | 1 |  |  |  | 1 | 2 |
| HSGS210\|asmbl_27589\|m.64242#B |  |  |  | 1 |  | 1 |
| HSGS210\|asmbl_30049\|m.73730 |  | 3 |  | 1 |  | 4 |
| HSGS210\|asmbl_31226\|m.77302 |  | 1 |  | 1 |  | 2 |
| HSGS210\|asmbl_34280\|m.88564 |  |  |  | 1 |  | 1 |
| HSGS210\|asmbl_36286\|m.95453 | 3 | 9 |  |  |  | 12 |
| HSGS210\|asmbl_36974\|m.97593 |  | 4 |  | 5 |  | 9 |
| HSGS210\|asmbl_42867\|m.116736 |  | 2 |  | 1 |  | 3 |
| HSGS210\|asmbl_44855\|m.123828 |  |  |  | 2 |  | 2 |
| HSGS210\|asmbl_45443\|m.125761 | 1 | 2 |  |  |  | 3 |
| HSGS210\|asmbl_7142\|m.137621 |  |  |  | 1 |  | 1 |
| HSGS210\|asmbl_8566\|m.141496 |  | 1 |  | 1 |  | 2 |
| HSGS210\|comp10435_c0_seq1\|m.150398 |  |  |  | 3 |  | 3 |
| HSGS210\|comp11043_c0_seq1\|m.151596 |  |  |  | 1 |  | 1 |
| HSGS210\|comp11909_c0_seq1\|m.153393 |  |  |  | 1 |  | 1 |
| HSGS210\|comp14684_c0_seq1\|m.165206#B |  |  |  | 1 |  | 1 |
| HSGS210\|comp15242_c0_seq2\|m.169384 |  |  |  |  | 1 | 1 |
