# Supplementary material for: Comparative genomics of Australian isolates of the wheat stem rust pathogen Puccinia graminis f. sp. tritici reveals extensive polymorphism in candidate effector genes
Source: Front Plant Sci. 2015 Jan 8;5:759. doi: 10.3389/fpls.2014.00759 (PMC4288056; doi:10.3389/fpls.2014.00759)
Supplement: Supplementary file 1 [file Data_Sheet_1.ZIP › Figures_S1 and_S2_30thSept.pptx]

## Slide 1
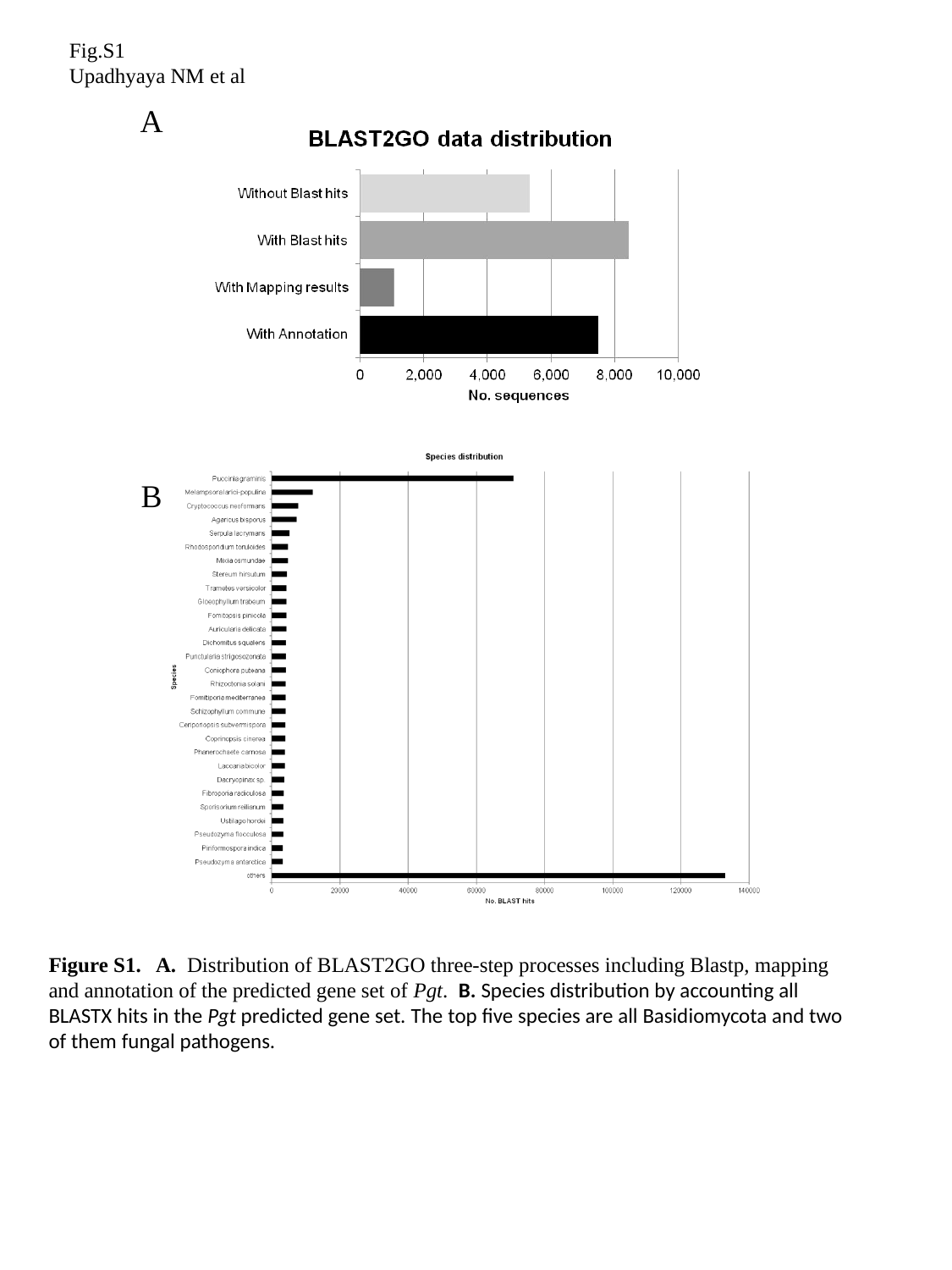

Fig.S1
Upadhyaya NM et al
A
Figure S1. A. Distribution of BLAST2GO three-step processes including Blastp, mapping and annotation of the predicted gene set of Pgt. B. Species distribution by accounting all BLASTX hits in the Pgt predicted gene set. The top five species are all Basidiomycota and two of them fungal pathogens.
B

## Slide 2
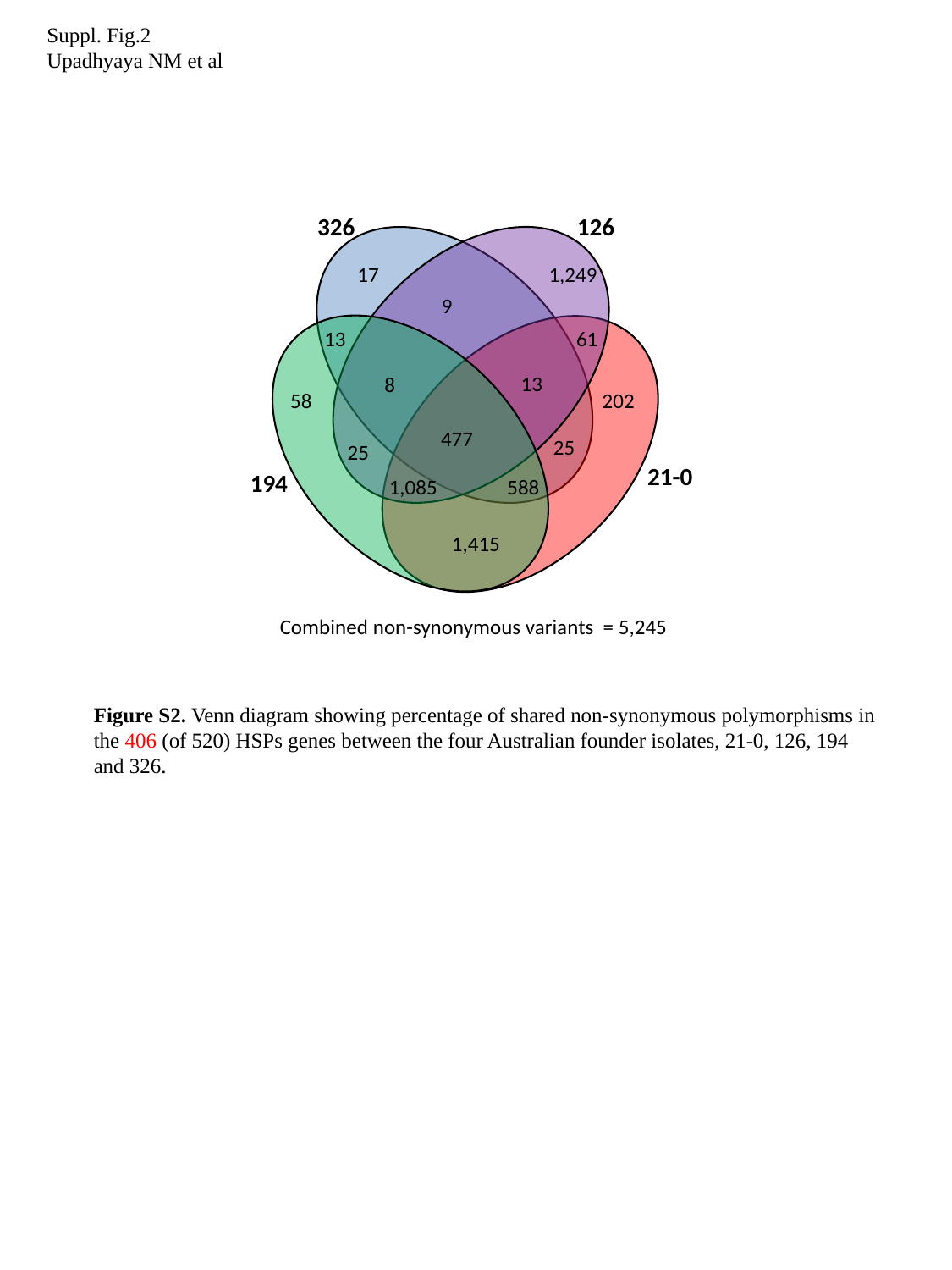

Suppl. Fig.2
Upadhyaya NM et al
326
126
1,249
17
9
13
61
13
8
58
202
477
25
25
21-0
194
588
1,085
1,415
Combined non-synonymous variants = 5,245
Figure S2. Venn diagram showing percentage of shared non-synonymous polymorphisms in the 406 (of 520) HSPs genes between the four Australian founder isolates, 21-0, 126, 194 and 326.
